# Everybody's Page

**Published:** 1888-03-10

**Authors:** 


### EVERYBODY'S PAGE.

THE Pasteur Institute, now in process of construction at Vangirard, will cost 1,500,000 francs.

DROWSY people have often been suffocated by resting the neck on a rope or the board at the head or foot of a bedstead.

A NEW absorbent has been made from cocoa-nut fibre, which will hold about twelve times its own weight of water.

LOOK upon a "sprain," though the name be short and homely Anglo-Saxon, as quite as important as the Latin "fracture"; it is by no means an uncommon occurrence to find that a sprain has proved the more important accident of the two.

A HAT, whether light or heavy, should be well ventilated. The scalp perspires as does the rest of the skin. In a badly-ventilated hat, the air within the hat and in contact with the head soon becomes fully charged with moisture; evaporation is in abeyance, the perspiration collects in visible amount, and the natural means for cooling the surface of the head are destroyed.

Do not consider pain as altogether an evil. It has important uses. It is to be regarded in the same way as you regard the red light on the railway, as a signal of danger. But having called attention to the fact that there is danger, the signal has done its duty and is dropped whenever the line is clear; in the same way, when pain has shown the need for rest and repose, it has fulfilled its part and must be got rid of, as after this it is only an evil.

A STRANGE case of complete mental collapse is exercising the minds of the medical staff at the Hotel Dieu, in Paris. Some months ago a woman of the working class, apparently about forty-five years old, and comfortably dressed, presented herself at a police station near the Odeon Theatre, and asked for shelter. As she did not appear to be in full possession of her mental faculties, she was sent to the Hotel Dieu, where she has remained ever since in a semi-comatose condition. Her memory does not go further back than the date of her application to the police station; it is quite a blank as to her name and her previous history.

MEDICAL men are devoted to the science of life—its preservation against the dangers which menace it through accident or disease—and they resent those stale old jokes which depict their profession as hand in hand with death. But the authorities of the State of New York have invited physicians, among others, to give information as to the best method of—not preserving life—but, on the contrary, inflicting death. While crime exists and capital punishment is the extreme penalty of the law, criminals must be executed; but the legislature of New York, recognising that the death of any human being, caused wilfully and deliberately by the law, is meant rather as a protection to the innocent members of society than as a punishment to the guilty one, have formed a committee to learn from judges, attorneys, physicians, and sheriffs, what mode of death they consider least open to objection. The unanimous answer is that the guillotine is too bloody, the garotte too barbarous, shooting too uncertain, and hanging too revolting, and that the best method of destroying life is by electricity, which gives instantaneous and painless death. The proposed method is that the criminal should be placed in a chair having metallic foot-rests connected with electrodes; and that a strong electric current should be passed through the body. It may be remembered that this manner of causing death is mentioned in Mr. Louis Stevenson's novel, *The Dynamiter*, where one of the characters commits suicide by means of an electric chair.

DR. COZZOLINO, lecturer on laryngology at the University of Naples, has introduced a new and very simple inhaler. This consists of a pair of metal tubes about three-quarters of an inch in length, and connected by a curved band. In these tubes are placed small pieces of blotting-paper saturated with whatever substance the patient is ordered to inhale. The tubes are placed in the nostrils, and the patient can then inspire by the nose and expire by the mouth till he has taken the whole dose of the volatile liquid in which the paper was soaked.

"TUNG-WAH" is the name of a philanthropic society at Hong-Kong. It is composed of the principal Chinese merchants of the colony, and represents the Chinese community, whose wishes are communicated to the Government through it. No Chinaman, it is said, has ever appealed to it in vain. This society has founded a large institution, known as the Hospital of Tung-Wah; which is, however, rather an asylum than a hospital, in which all subjects of the Middle Kingdom may receive assistance, whether they are ill or not. About ten years ago Tung-Wah established a branch organisation named "Po Seung kuk," for the protection of women and children. Both these institutions were supported and encouraged during his administration by Sir John Pope Hennessey, who was distinguished among the English residents of Hong-Kong by his numerous acts of philanthropy towards the Chinese.

THE Berlin Anthropological Museum has just received a present of a very valuable and interesting nature through the generosity of a Parsee banker, Sir Jamsadji Jijibhai. It is a model of one of the "towers of silence," in which the Parsees bury their dead. According to the religion of the Zoroastrians the three sacred elements—fire, earth, and water—must not be desecrated by dead bodies, which are, therefore, given to birds of prey. For this purpose high round towers, which have a circumference of 300 feet, are built on the hills. At the top of the tower is a platform sloping towards the centre, where there is placed a cistern 150 feet deep. The platform is divided into three equal parts, set aside for the bodies of men, women, and children respectively. As soon as the corpses have been placed on the platform flocks of vultures, which are always hovering around, begin their ghastly meal, and before long only the bare bones are left. These are swept into the cistern by means of water, with which the platform is flushed, and after the bones are dissolved in the cistern the water passes through four subterranean canals, and is then disinfected by means of a filter.

PROFESSOR RAY LANKESTER, speaking at the London Institution, said "the future of preventive medicine is the education of the white blood corpuscle." What is the meaning of this seemingly mysterious utterance? This: White corpuscles are small protoplasmic cells floating in the blood, whose mission is to consume any poisonous elements they meet with there. They gather in battle array against any dangerous bacteria, and fight till one force or the other is destroyed. Sometimes they saturate themselves with the poisonous particles from a wound, and then eject themselves from the body. But the white corpuscle can absolutely feed on poison, and thereby grow stronger to consume more. The theory of vaccination is based on this function of the white corpuscle. The corpuscle which has been reared on vaccine lymph can devour without injury the more dangerous virus of small-pox. This explains also how confirmed consumers of opium, arsenic, and other poisons can take with impunity doses of these drugs which would kill the uninitiated. But may we not train our corpuscles to better uses than this?

